# Immunologic Drivers and Restraints in Colitis-Associated Colorectal Cancer

**DOI:** 10.3390/cancers18081230

**Published:** 2026-04-13

**Authors:** Rachele Frascatani, Federica Laudisi, Carmine Stolfi, Giovanni Monteleone

**Affiliations:** 1 Department of Systems Medicine, University of “Tor Vergata”, 00133 Rome, Italy; rakfrasc@gmail.com (R.F.); federica.laudisi@uniroma2.it (F.L.); carmine.stolfi@uniroma2.it (C.S.); 2 Gastroenterology Unit, Policlinico Universitario Tor Vergata, 00133 Rome, Italy

**Keywords:** IBD, ulcerative colitis, tumor immunosurveillance, cytokines, immune cells

## Abstract

Inflammatory bowel diseases (IBD), encompassing ulcerative colitis and Crohn’s disease, are associated with an increased risk of colorectal cancer through mechanisms driven by persistent mucosal inflammation. Chronic inflammatory signaling, recurrent epithelial injury, and altered tissue repair processes progressively reshape the intestinal microenvironment, promoting genomic instability and facilitating the development of colitis-associated colorectal cancer (CAC). Despite the well-established link between inflammation and tumorigenesis, only a subset of patients with long-standing IBD develops malignancy, highlighting the complexity of the regulatory effects of the ongoing inflammation on the tumor initiation and progression. This review discusses the multifaceted roles of innate and adaptive immune responses in CAC pathogenesis. Innate immune signaling mediated by pattern recognition receptors, particularly Toll-like receptors, integrates microbial and damage-associated signals to activate inflammatory pathways that regulate epithelial proliferation, survival, and tumor-promoting cytokine networks. Tumor-associated macrophages, neutrophils, and myeloid-derived suppressor cells contribute to carcinogenesis by sustaining chronic inflammation, promoting immunosuppression, and remodeling the tumor microenvironment, although under specific conditions these cells can also support antitumor immunity. Innate lymphocyte subsets participate in immune surveillance and epithelial homeostasis, yet may also amplify inflammatory circuits that influence tumor development. Adaptive immune populations further shape CAC evolution, as CD4^+^ T-helper subsets, CD8^+^ cytotoxic T lymphocytes, regulatory T cells, and B cells exert divergent effects depending on cytokine milieu, immune context, and disease stage. Understanding immune-cell plasticity and the molecular pathways governing these processes may facilitate the identification of predictive biomarkers and the development of targeted immunomodulatory strategies aimed at preventing CAC.

## Simple Summary

Chronic intestinal inflammation is a key driver of colorectal carcinogenesis in patients with inflammatory bowel diseases (IBD), including ulcerative colitis and Crohn’s disease. Persistent epithelial injury, dysregulated immune signaling, and repeated cycles of tissue damage and repair reshape the intestinal microenvironment, promoting genomic instability and increasing the risk of colitis-associated colorectal cancer (CAC). However, only a subset of patients with long-standing IBD develops malignancy, suggesting that immune responses within the inflamed mucosa play complex and context-dependent roles in tumor initiation and progression. Moreover, inflammation is not universally associated with tumorigenesis. Under specific immune contexts, certain immune cell subsets activate potent antitumor pathways capable of recognizing and eliminating dysplastic or early malignant cells, thereby restraining carcinogenesis. Understanding the factors that shift inflammation from protective to pathogenic remains essential for deciphering the pathogenesis of CAC. This review examines the dualistic influence of gut inflammatory cells on CAC initiation and progression, highlighting both their detrimental and protective roles.

## 1. Introduction

Chronic inflammation is now widely recognized as a fundamental driver of carcinogenesis across multiple organ systems, acting through sustained tissue injury, aberrant repair mechanisms, and persistent immune activation [1]. This relationship is particularly evident in inflammatory bowel diseases (IBD), encompassing ulcerative colitis (UC) and Crohn’s disease (CD), where long-standing inflammation profoundly alters the epithelial compartment [2]. Recurrent epithelial damage, excessive cytokine signaling, and dysregulated immune–epithelial interactions create conditions that favor genomic instability and malignant transformation, thereby predisposing affected individuals to colorectal cancer (CRC) [3,4,5]. In IBD, CRC risk is strongly associated with both the extent of intestinal involvement and the duration of disease, underscoring the concept that cancer risk reflects the integrated inflammatory burden over time rather than isolated disease flares [6,7,8,9,10,11]. Epidemiological studies consistently demonstrate that colitis-associated CRC (CAC) risk increases progressively after several years of active disease [12,13], forming the rationale for surveillance colonoscopy programs that typically begin approximately eight years after symptom onset to detect dysplasia and early neoplastic lesions at a curable stage [14,15,16,17].

In general, the incidence rates of CAC vary from those observed in sporadic CRC. In the United Kingdom, the cumulative incidence of CAC was reported as 0.1% after 10 years, 2.9% after 20 years, and 6.7% after 30 years [18]. Similar trends were documented in Asian countries, with a pooled prevalence of CAC estimated at 0.85% [19,20]. A recent meta-analysis that included 70 studies and 637,414 participants reported overall prevalence rates of adenomas, advanced adenomas, and sporadic CRC at 23.9%, 4.6%, and 0.4%, respectively [21].

Large population-based cohorts have reported increased detection of dysplasia in recent decades, even though the incidence of CAC appears to be declining among patients enrolled in surveillance programs [20,22]. This apparent paradox likely reflects multiple converging factors, including advances in medical therapy that effectively reduce chronic inflammatory activity, widespread use of maintenance treatment, and improved endoscopic techniques [23,24,25]. It is also plausible that the observed reduction in CAC incidence results, at least in part, from the true protective impact of structured surveillance [26]. Despite these advances, it remains unclear why only a subset of patients with long-standing IBD ultimately develop CAC, while others with comparable disease duration and extent do not [12,27,28]. Additionally, it is uncertain whether the observed decrease in CAC incidence in recent years will be affected by the marked reduction in the number of colonoscopies performed during the COVID-19 pandemic, which has led to significant disruptions in routine surveillance schedules [29,30].

Immune cells infiltrating the chronically inflamed intestinal mucosa play complex roles in shaping cancer risk [31]. Rather than acting uniformly as tumor promoters, immune populations can either restrain or facilitate neoplastic progression depending on their functional state, spatial localization, and cytokine output [32,33]. Type 2 macrophages, regulatory T cells, and myeloid-derived suppressor cells (MDSC) are frequently associated with immunosuppressive or pro-tumorigenic environments that favor epithelial transformation and tumor growth [34,35,36]. In contrast, cytotoxic CD8^+^ T lymphocytes and natural killer (NK) cells are generally linked to effective immune surveillance and tumor cell elimination, contributing to the containment of malignant expansion [37,38]. Much of this regulatory influence is mediated by cytokines, which act on transformed cells to regulate proliferation, survival, differentiation, and DNA damage responses [39]. For a comprehensive overview of how specific cytokines contribute to colorectal tumorigenesis, readers are referred to several detailed articles and reviews on this topic [40,41,42,43,44,45]. The main promoting or suppressive effects of immune cell-derived cytokines on the initiation and progression of colon tumorigenesis are summarized in Table 1.

Beyond direct immune–epithelial interactions, immune cells also shape carcinogenesis indirectly by activating stromal fibroblasts and endothelial cells, thereby promoting the release of growth factors, angiogenic mediators, and extracellular matrix components that support tumor initiation and progression [42,46,47,48,49].

**Figure 1:**
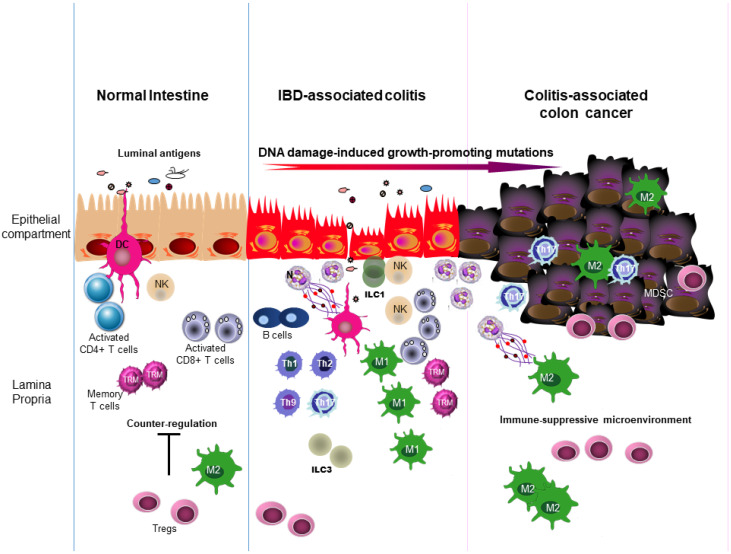
Schematic view showing the involvement of immune cells in the evolutionary stages that lead from a normal intestine to inflammatory bowel disease (IBD)-associated colitis and colitis-associated cancer (CAC). The normal intestine is infiltrated by activated immune cells, the function of which is tightly regulated by counter-regulatory cells (e.g., T-regulatory cells [Tregs], type 2 macrophages [M2], and myeloid-derived suppressor cells [MDSCs]). In IBD, the mucosa is massively infiltrated by various immune cells, which are not adequately controlled by regulatory cells. Additionally, the phenotype of some immune cells (e.g., macrophages) differs from that of the same cell types present in the normal intestine and in CAC. In the latter, the presence of many regulatory cells promotes the induction of an immune-suppressive microenvironment that sustains tumor growth. Abbreviations: DC, dendritic cells; M, Macrophages; MDSC, Myeloid-Derived Suppressor Cells; NK, Natural Killer cells; Tregs, Regulatory T Cells; ILC, innate lymphoid cells; TREM, memory T cells; Th, T helper cells; N, neutrophils.

These observations highlight that inflammation-driven carcinogenesis in IBD is not simply a consequence of immune activation, but rather reflects a dynamic imbalance between protective immune surveillance and pathogenic immune-mediated tissue remodeling (Figure 1).

In this review, we examine the dual and context-dependent roles of intestinal inflammation in the development of CAC, with particular emphasis on immune-cell plasticity, namely the ability of immune cells to change their function, phenotype, or behavior in response to different environmental signals, within the tumor microenvironment. Understanding which immune pathways are protective and which are permissive for malignancy may ultimately enable more precise surveillance approaches and immune-modulating therapies that reduce cancer risk without compromising mucosal defense.

## 2. Dual Role of Immune Cells in IBD-Associated CRC

Despite significant advances in experimental modeling of CAC, it remains difficult to clearly distinguish intestinal inflammatory responses that promote tumor development from those that suppress it [50,51]. One key challenge stems from the remarkable plasticity of immune cell populations, which enables them to alter their functional behavior in response to local environmental stimuli, microbial products, metabolic stress, and epithelial damage [33,52,53]. In IBD, this adaptability is further intensified by the chronic, relapsing course of inflammation [54]. While endogenous anti-inflammatory mechanisms are essential for restoring tissue integrity and maintaining homeostasis, they may also create an immunosuppressive microenvironment, which can weaken immunosurveillance and ultimately support the survival and expansion of dysplastic cells [35,51,55,56,57]. Moreover, the recurrent inflammatory episodes continuously remodel cytokine networks, chemokine gradients, and metabolic conditions within the gut mucosa [58,59,60,61,62]. Consequently, a single immune cell subset may play contrasting roles at different stages of carcinogenesis, from tumor initiation to progression and eventual dissemination [61]. These considerations highlight that the functional impact of immune responses in CAC cannot be understood in static terms but must instead be interpreted within the dynamic context of disease stage, microenvironmental signals, and immune-cell plasticity.

While the involvement of immune cells in CAC has been discussed in earlier reviews, the following sections focus on the distinct roles played by specific subsets of innate and adaptive immune cells in this process. We highlight how immune context and timing influence CAC development, as well as the potential for modulating immune responses in the prevention and treatment of CAC.

## 3. Gut Microbiota and Toll-like Receptor Signaling in Colitis-Associated Colon Cancer

CAC is not only tightly connected to chronic inflammation but also strongly associated with dysbiosis, which arises from changes in the composition of the gut microbiota [63,64]. In the context of an inflammatory intestinal environment, pathogenic bacteria and their metabolites can influence tumorigenesis either by directly damaging the epithelial cells, triggering oncogenic signaling, and causing DNA damage, or by modulating the activation and function of immune cells, thereby affecting the persistent chronic inflammation [65,66]. For example, Enterotoxigenic Bacteroides fragilis produces the pathogenic Bacteroides fragilis toxin, which binds to receptors on colonic epithelial cells, triggering the Wnt and NF-κB signaling pathways. This activation results in enhanced epithelial cell proliferation, the release of pro-inflammatory mediators, and DNA damage. Additionally, it compromises the intestinal barrier, thereby enhancing the ongoing inflammation and, eventually, tumorigenesis [67,68]. The prevalence of *Escherichia* (*E.*) *coli* containing the polyketide synthase (pks) genomic island is notably higher in the tissues of patients with IBD and CRC compared to healthy individuals, indicating a possible involvement in the development of intestinal lesion [69]. pks+ *E. coli* has been shown to cause DNA damage in host epithelial cells and to induce functional mutations linked to the p53 and Wnt signaling pathways when human colon organoids are exposed to pks+ *E. coli* in an acute setting, thereby elevating the risk of CRC [70].

*Fusobacterium* (*F.*) *nucleatum* is an invasive, adhesive, and pro-inflammatory anaerobic bacterium that tends to accumulate in the intestines of individuals with IBD or CRC, displaying carcinogenic characteristics [71]. *F. nucleatum* worsens colitis by compromising epithelial integrity and influencing the polarization of M1 macrophages [72]. However, unlike other bacteria linked to colon tumorigenesis, *F. nucleatum* does not exacerbate CAC.

At the same time, the loss of protective commensal bacteria, like Lactobacillus and Bifidobacterium, might play a significant role in the tumorigenic process due to their ability to inhibit the colonization of gut pathogens, maintain microbial balance, regulate intestinal transit time and short-chain fatty acid production, and support mucosal barrier integrity. In line with this, administration of Lactobacillus rhamnosus has been shown to decrease tumor number and size in the AOM+DSS-induced CAC mouse model. Lactobacillus rhamnosus also restored the key genus-level alterations in the gut microbiota caused by AOM/DSS treatment [73].

Pattern Recognition Receptors (PRRs) are key components of the innate immune system, responsible for detecting pathogen-associated molecular patterns (PAMPs) found in microorganisms and damage-associated molecular patterns (DAMPs) released from dying or injured cells [62]. Engagement of PRRs triggers downstream signaling cascades that activate transcription factors such as nuclear factor kappa B (NF-κB), leading to the expression of pro-inflammatory cytokines, adhesion molecules, and extracellular matrix regulators [74]. These molecules facilitate immune cell recruitment and activity within the local tissue microenvironment, playing critical roles in the regulation of tumor initiation and progression [73]. Among the PRR family, Toll-like receptor 4 (TLR4) stands out due to its broad range of ligands, which include both endogenous DAMPs and exogenous PAMPs (i.e., Gram-negative bacteria-derived lipopolysaccharide) [75,76]. TLR4 is overexpressed in colonic epithelial cells of UC patients and in human and murine CAC [77]. Upon activation, TLR4 triggers the upregulation of cyclooxygenase-2, stimulates the production of prostaglandin E_2_, and activates epidermal growth factor receptor signaling pathways [78]. This cascade of events contributes to increased cell proliferation, enhances the survival of malignant cells by inhibiting apoptosis, and promotes tumor invasion and metastasis [77,79]. Blockade of TLR4 signaling during the inflammatory phases of azoxymethane (AOM)+ dextran sulfate sodium (DSS)-induced CAC reduces both the development and progression of colonic tumors [77,80]. This finding is associated with a decrease in the number of infiltrating macrophages and lower levels of colonic pro-inflammatory cytokines compared to control mice. Notably, inhibiting bacterial signaling through antibiotic treatment during the inflammatory phases of CAC also protects mice from colitis and nearly completely prevents tumor growth [80]. These results suggest that bacterial activation of TLR4 in the colon drives inflammation and facilitates tumor progression. On the other hand, there is evidence that TLR4-deficient mice are largely protected against the development of tumors as compared to wild-type mice [77]. Consistent with this, mice lacking E3 ubiquitin–protein ligase Pellino homolog 3 (Pellino 3), a RING-type E3 ubiquitin ligase, show reduced activation of inflammatory signaling during the early stages of carcinogenesis after AOM+DSS treatment. Mechanistic studies suggest that Pellino 3 promotes TLR4-mediated inflammation by facilitating the ubiquitination-dependent degradation of interferon regulatory factor 4 (IRF4), a negative regulator of TLR4 in macrophages [81]. It has also been proposed that miR-155 may enhance TLR4 signaling by targeting negative regulators of TLR4, including Suppressor of Cytokine Signaling 1 and Src homology 2 domain-containing inositol polyphosphate 5-phosphatase 1 [82,83]. Interestingly, TLR4 activation induces the expression of miR-155 through both transcriptional and post-transcriptional mechanisms [84]. This creates a positive feedback loop between TLR4 and miR-155, which could potentially accelerate the development of CAC [83].

Contrarily, signaling mediated by myeloid differentiation factor 88 (MyD88), a molecule critical for TLR and receptors for the proinflammatory cytokines of the interleukin (IL)-1 family [85], exerts divergent effects during the development and progression of AOM+DSS-induced CAC. For instance, MyD88-deficient mice have defective ulcer healing following epithelial injury, which is associated with changes in the expression of genes involved in pro-inflammatory responses, cell proliferation, apoptosis, and DNA repair pathways. Consequently, these mice display a marked increase in adenoma incidence and progression toward invasive adenocarcinomas, frequently accompanied by clonal mutations in the β-catenin gene [86,87]. In contrast, treating wild-type mice with TJ-M2010-5, an inhibitor of MyD88 signaling, results in the depletion of MDSCs, effectively preventing AOM+DSS-induced CAC [88].

Additional TLR family members contribute to CAC regulation. For example, TLR3, which recognizes viral double-stranded RNA, has been shown to exert protective effects in experimental colitis and CAC models [89]. Activation of TLR3 signaling using polyinosinic–polycytidylic acid, a synthetic agonist of TLR3, reduces the severity of DSS-induced acute colitis in wild-type mice [90]. Consistent with this, TLR3-deficient mice show enhanced colitis and an increased tumor burden after treatment with AOM and DSS [91].

TLR13 recognizes a conserved sequence within bacterial 23S ribosomal RNA and contributes to host defense against pathogenic bacteria [92]. Loss of TLR13 exacerbates colitis severity and promotes AOM+DSS-induced CAC development through enhanced intestinal permeability, prolonged production of inflammatory cytokines such as IL-6, IL-12, and tumor necrosis factor-α (TNF-α), and increased signaling through STAT3, NF-κB, and MAPK pathways [93].

The divergent effects of TLRs on the development of CAC are, at least in part, dependent on the different ability of such receptors to activate specific signaling pathways. Therefore, the TLR4-driven pro-tumorigenic effects are mediated by activation of both NF-kB and Mitogen-Activated Protein Kinase (MAPK) and subsequent production of pro-tumorigenic cytokines (e.g., IL-6, TNF-α), while the TLR3 and TLR13-dependent tumor suppression would rely on IRF3 signaling and downstream production of type I interferon [77,78,79,80,91].

Collectively, these findings underscore the complexity of innate immune regulation in CAC and suggest that selective modulation, rather than broad inhibition, of innate immune signaling pathways may represent a more effective strategy for preventing CAC.

## 4. Tumor-Associated Macrophages

Tumor-associated macrophages (TAMs) represent one of the most abundant and functionally versatile immune populations within the microenvironment of CAC. These cells exert opposing roles in the development and progression of CAC, partly depending on their capacity to produce cytokines, which can shape the tumor microenvironment in ways that favor malignant transformation or, conversely, enhance antitumor immune responses [34].

**Figure 2:**
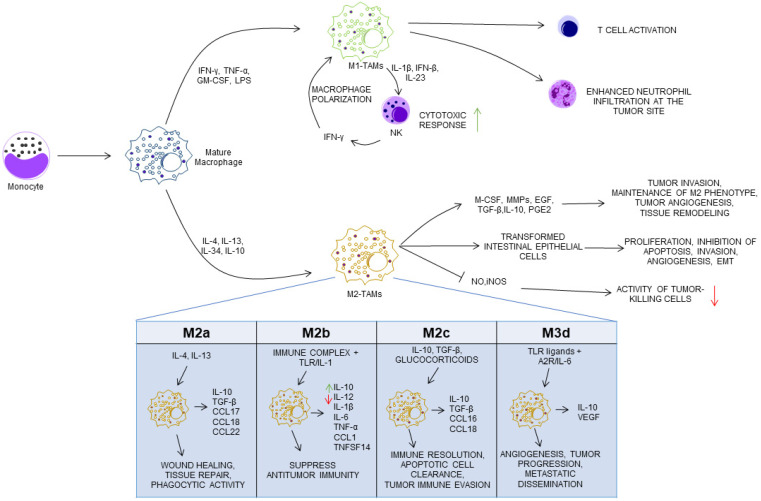
Macrophage polarization and functional roles in colitis-associated colon cancer (CAC). Intestinal macrophages originate from circulating monocytes and undergo differentiation in response to microenvironmental signals. Pro-inflammatory stimuli (e.g., LPS, IFN-γ, TNF-α, GM-CSF) promote M1 polarization, whereas IL-4, IL-13, IL-34 and IL-10 induce M2 differentiation. M1-TAMs exert antitumor effects by producing pro-inflammatory cytokines, enhancing NK and T-cell responses, and promoting neutrophil recruitment. In contrast, M2-TAMs support tumor progression by secreting anti-inflammatory and pro-tumorigenic factors that promote proliferation, angiogenesis, invasion, and immune suppression. M2 macrophages include distinct subsets (M2a–d), which vary in their ability to produce different cytokines/chemokines and, consequently, in their roles in tissue remodeling, immune regulation, and tumor progression. Abbreviations: A2R, Adenosine A2 Receptor; EGF, Epidermal Growth Factor; GM-CSF, Granulocyte-Macrophage Colony-Stimulating Factor; IFN beta, Interferon-β; IFN gamma, Interferon-γ; iNOS, Inducible Nitric Oxide Synthase; LPS, Lipopolysaccharide; M-CSF, Macrophage Colony-Stimulating Factor; M1-TAMs, M1 Tumor-Associated Macrophages; M2-TAMs, M2 Tumor-Associated Macrophages; MMPs, Matrix Met-alloproteinases; NK, Natural Killer cells; NO, Nitric Oxide; PGE2, Prostaglandin E2; TGF beta, Transforming Growth Factor-β; TLR, Toll-Like Receptors; TNF alpha, Tumor Necrosis Factor-α; TNFSF14, Tumor Necrosis Factor Superfamily Member 14; VEGF, Vascular Endothelial Growth Factor.

Traditionally, macrophages have been classified into two broad polarization states: classically activated M1 macrophages and alternatively activated M2 macrophages. M1 macrophages typically arise in microenvironments enriched in TLR ligands and pro-inflammatory cytokines. These cells produce high levels of TNF-α, IL-1β, IL-6, IL-12, IL-23, and type I interferons, thereby promoting cytotoxic immune responses mediated by NK cells and CD8^+^ T lymphocytes [34,94,95,96]. IL-12, in particular, drives the differentiation of T helper (Th1) cells, which secrete interferon-γ (IFN-γ) and further reinforce M1 macrophage polarization through positive feedback mechanisms (Figure 2) [97].

Consequently, excessive M1 macrophage activation is believed to aggravate the mucosal inflammatory response in IBD while simultaneously supporting antitumor immune surveillance [98]. Consistent with this concept, treatment with diphenyleneiodonium (DPI), an inhibitor of NADPH oxidase, reduces M1 macrophage polarization, ameliorates DSS-induced colitis, and attenuates tumorigenesis in the AOM/DSS model of CAC [99]. In addition to sustaining inflammatory responses, M1 macrophages can exert direct antitumor effects. For example, these cells can induce the expression of tumor necrosis factor (TNF)-related apoptosis-inducing ligand (TRAIL) on tissue stem cells, thereby promoting apoptosis of transformed epithelial cells and reducing tumor formation in experimental CAC [100]. Furthermore, M1 macrophages release chemokines that recruit neutrophils and other effector immune cells to sites of inflammation, amplifying acute immune responses that may contribute to tumor cell elimination [34].

Alternatively activated M2 macrophages emerge in response to glucocorticoids, immune complexes, lipopolysaccharide, Th2-associated cytokines (e.g., IL-4, IL-10, and IL-13), or IL-34 signals [47]. These macrophages are further subdivided into M2a, M2b, M2c, and M2d subsets, each characterized by distinct activation signals, effector molecules, and biological functions [101]. M2a macrophages, induced by IL-4 and IL-13, secrete IL-10, transforming growth factor-β (TGF-β), and chemokines such as CCL17, CCL18, and CCL22, promoting tissue repair and fibrosis [94,95,96]. M2b macrophages arise following immune-complex engagement combined with TLR or IL-1 receptor signaling and exhibit a mixed cytokine profile, characterized by high IL-10 and low IL-12 production, which strongly suppresses effective antitumor immunity [102,103]. M2c macrophages, driven by IL-10, TGF-β, or glucocorticoids, play key roles in immune resolution and clearance of apoptotic cells but may also facilitate tumor immune evasion [104,105,106].

M2d macrophages, often considered the prototypical TAM population, are induced by IL-6 or adenosine receptor signaling and support angiogenesis, tumor growth, and metastatic dissemination (Figure 2) [107,108].

Although the above data indicate that macrophage polarization can affect the occurrence of CAC by regulating either the mucosal inflammation or the tumor microenvironment, this binary classification oversimplifies the remarkable heterogeneity and plasticity of macrophages within inflamed and neoplastic intestinal tissue. Environmental factors including hypoxia, microbial metabolites, epithelial damage signals, and micronutrient availability can actively reprogram macrophage function [109,110,111,112]. For instance, ornithine decarboxylase (Odc), a key enzyme in polyamine biosynthesis, suppresses M1 activation and is upregulated in colonic macrophages from patients with active IBD, dysplasia, and CAC. Genetic deletion of Odc in myeloid cells reduces colitis severity and tumor burden in experimental models, highlighting the importance of metabolic regulation in macrophage-driven carcinogenesis [113]. Similarly, nicotinamide phosphoribosyltransferase (NAMPT), a central enzyme in NAD^+^ metabolism, has been shown to stabilize the M2 phenotype in sporadic CRC by sustaining HIF-1α signaling and dampening interferon responses [109]. The complement component C6 also modulates macrophage polarization, as C6 deficiency enhances M2 responses and worsens CAC in AOM+DSS-treated mice, accompanied by increased CCL2 and CXCL13 and reduced CCL17 [110]. Additional control is exerted by Pellino1, which is upregulated in UC, CAC, and murine colitis models [81,111]. Monocyte-specific deletion of Pellino1 reduces M2 recruitment and attenuates colitis and CAC. Mechanistically, Pellino1 ubiquitinates STAT3 (K63-linked), driving pathogenic STAT3 activation [111]. Spatial transcriptomic and immunophenotyping analyses indicate that the non-inflamed colon of IBD patients who later develop CAC exhibits upregulated metabolic and stress–response pathways compared with sporadic CRC, suggesting ongoing epithelial stress. The overall immune cell density in the colonic lamina propria of these patients remained unchanged, but the chronic inflammation drives a suppressive phenotype characterized by increased IL-10 expression by IgA^+^ plasma cells and CD163^+^ macrophages [112].

The natural flavonoid vitexin can reprogram M2-type TAMs toward a pro-inflammatory, M1-like state through activation of the vitamin D receptor (VDR) and the VDR/PBLD pathway, delaying the transition from colitis to carcinoma [114]. This observation is particularly relevant given the frequent vitamin D deficiency observed in patients with IBD and CAC [115,116]. Experimental models show that VDR downregulation accompanies colitis onset and promotes the formation of larger and more numerous tumors [114]. Additionally, induction of ferroptosis in macrophages can shift TAMs toward an M1 phenotype [117].

Collectively, these findings suggest that macrophage polarization represents a critical, targetable determinant of cancer risk in IBD and that therapeutic strategies aimed at restoring macrophage balance may offer translational opportunities for CAC prevention.

## 5. Neutrophils, Myeloid-Derived Suppressor Cells and Eosinophils

Neutrophils, once considered short-lived bystanders of acute inflammation, are now recognized as central drivers of chronic intestinal inflammation and contributors to the development of CAC [118,119]. In IBD, numerous disease-associated genetic variants affect neutrophil functions, particularly those involved in ROS production and microbial killing (e.g., CYBA, CYBB, NCF4, IFNGR2) [120]. The inflamed intestinal mucosa of patients with active disease is characterized by extensive neutrophil infiltration, driven by elevated levels of chemokines such as IL-8, CXCL5, CXCL7, CXCL10, and CCL20, as well as delayed neutrophil apoptosis [121]. Activated neutrophils release large quantities of proteolytic enzymes, including neutrophil elastase and matrix metalloproteinases [122]. While these mediators are essential for host defense, their sustained release promotes epithelial cell injury, disrupts barrier integrity, and facilitates transepithelial migration, leading to characteristic histopathological features of IBD such as cryptitis and crypt abscesses [123]. In addition, neutrophils act as potent amplifiers of inflammation by secreting cytokines and chemokines (e.g., IL-1β, IL-8, IL-17, TNF, CXCL1, CXCL2, CXCL5) that recruit and activate other immune cells, including monocytes, DC, NK cells, and T cells, thereby perpetuating inflammatory circuits that could favor tumor initiation and progression [121]. UC patients with significant mucosal neutrophil infiltration are at significantly higher risk of developing CAC [119,124], raising the possibility that neutrophils might be important drivers of the transition from chronic IBD to CAC. Neutrophil-derived ROS could be implicated in the development of CAC due to their ability to modulate major signaling pathways (e.g., NF-κB, Src kinase, HIF, PI3K/Akt/mTOR, RAS/ERK, and JNK/p38) that enhance resistance to apoptosis, and favor epithelial–mesenchymal transition and angiogenesis [125]. Neutrophil-derived serine proteases suppressed FOXA2 expression, causing dysfunction of autophagy and eventually promoting AOM+DSS-induced CAC [126]. ChemR23, a G-protein-coupled receptor activated by resolvin E1, is upregulated in the inflamed colonic mucosa of patients with IBD and is associated with increased neutrophil accumulation. In murine models, treatment with an agonistic anti-ChemR23 antibody, which stimulates receptor signaling, enhances macrophage efferocytosis, decreases mucosal neutrophil infiltration, accelerates the resolution of colitis, and ultimately suppresses the development of CAC, further supporting the role of neutrophils in the CAC development [127].

Neutrophils express high levels of the histamine receptor 2, the signaling of which dampens the proinflammatory responses of mature neutrophils. Consistent with this, blockade of histamine receptor 2 inhibits myeloid maturation and the accumulation of CD11b+Ly6G+ immature myeloid cells, thus accelerating the progression of inflammation-associated colonic tumorigenesis and enhancing the infiltration of neutrophils into both inflamed tissue and CAC sites [128,129].

A distinctive feature of neutrophil activation in chronic inflammation is the formation of neutrophil extracellular traps (NETs), composed of chromatin fibers decorated with histones, proteases, and granular proteins [130]. During their formation, NETs not only activate proinflammatory factors like myeloperoxidase (MPO) and neutrophil elastase but also trigger the activation of peptidylarginine deiminase type 4 (PAD4) [131]. PAD4 catalyzes the citrullination of histones, especially histone H3, leading to chromatin depolymerization and structural loosening. This process triggers nuclear and plasma membrane rupture, facilitating NETosis [132]. Following cell lysis, DNA, citrullinated histones, and other intracellular components are released, together forming extracellular traps [130].

In the gut, NETs may exert context-dependent effects, contributing either to microbial containment or to tissue injury and immunothrombosis [133]. Although their role in CAC remains incompletely defined, studies in sporadic CRC raise the possibility that excessive or dysregulated NET formation in IBD may contribute to malignant transformation by altering immune cell recruitment and function within the tumor microenvironment [134,135]. For instance, NETs could induce monocytes to release pro-tumorigenic cytokines [130], and the partially high concentration of MPO from NETs would also cause an oxidative stress response in epithelial cells, aggravating DNA damage and mutation [136]. Elevated NET levels have been detected in both the tissue and circulating blood of CRC patients, and such increases correlate with poorer outcomes in individuals undergoing radical surgical resection [137,138]. In murine models, NETs promote the capture and dissemination of platelet–tumor cell aggregates, thereby enhancing metastasis [139]. The pro-tumorigenic effects of NETs are indirectly supported by studies investigating the anti-cancer properties of Huang Qin Decoction (HQD), a traditional Chinese medicine commonly used in the treatment of colitis [140]. In the AOM/DSS-induced CAC mouse model, HQD specifically reduced the number of tumors, an effect linked to decreased neutrophil infiltration in the colon and enhanced CD8+ T cell immunosurveillance. Protein–protein interaction analysis indicated that HQD-mediated inhibition of CAC was associated with the deactivation of PAD4, which led to the downregulation of NETs, MPO-DNA complex levels, and PAD4 expression following HQD treatment [141]. In line with this, Glycyrrhizic acid (GA), a natural compound extracted from licorice, alleviated colitis and reduced tumor development in mice treated with AOM+DSS. The reduced tumorigenicity seen in mice receiving GA was accompanied by decreased NET formation, as evidenced by lower levels of PAD4, citrullinated histone H3, and MPO. In vitro studies revealed that GA effectively bound to and inhibited the activity of PAD4 [142].

NETs could also indirectly support tumor progression and metastasis by promoting angiogenesis, suppressing cytotoxic T-cell infiltration and activity [143], skewing macrophages toward the M2 phenotype, and facilitating the recruitment of MDSCs [144], a heterogeneous population of immature myeloid cells resembling dysfunctional neutrophils, which are enriched in the mucosa of IBD patients [145] as well as in CAC lesions, the latter process being regulated by GM CSF [120]. Depletion of MDSCs attenuates the progression from experimental colitis to CAC [146]. Studies in CAC models developing in STAT1-deficient mice showed that blockade of IL-17 attenuates the tumor formation and reduces the recruitment of neutrophils into intestinal tissue, and the expression intestinal STAT3, and of Arginase-1 and inducible nitric oxide synthase in the colon, both associated with the main suppressive activity of MDSCs [147]. Furthermore, the inhibition of neutrophil chemokines has been shown to impede disease progression in CAC model mice. In the same models, targeting neutrophil and MDSC-derived chemokines attenuates the development of CAC [148,149,150]. Importantly, the intestinal microbiota further enhances the immunosuppressive capacity of MDSCs, linking dysbiosis to immune escape and tumor progression [151]. Although this remains to be verified, these data suggest that neutrophil and MDSC activity might serve as biomarkers of cancer risk in IBD.

Eosinophils have been traditionally recognized as terminally differentiated, end-stage granulocytes that play a cytotoxic role in defending against parasitic helminths. After maturing in the bone marrow, eosinophils enter the circulation in response to IL-5, and possibly GM-CSF, before migrating to the gastrointestinal tract and other peripheral tissues, including the thymus, uterus, and mammary glands under normal condition [152]. In the gut, eosinophils also act as regulators of inflammation, epithelial barrier maintenance, and tissue remodeling [153,154]. Activated eosinophils accumulate in the gut of IBD patients, where their degranulation leads to a massive release of both cytotoxic proteins and pro-inflammatory cytokines, thus contributing to the epithelial barrier disruption [155]. Eosinophil accumulation is also a hallmark of cancer-related inflammation, probably reflecting the dominant production of Th2-type cytokines (e.g., IL-5) [154]. In sporadic CRC, the accumulation of eosinophils within tumors has been associated with a better prognosis and improved patient survival [156]. Less is known about the role of eosinophils in CAC, although indirect evidence suggests they might play a dual role. For example, mice deficient in IL-33, a potent eosinophil activator that stimulates their degranulation [157], exhibit gut microbiota dysbiosis and are highly susceptible to both colitis and CAC [158]. These data align with the demonstration that IL-33 administration reduces the tumor growth in AOM+DSS-induced CAC through a process that requires the presence of eosinophils [159]. On the other hand, mice deficient in CCL11, also known as eotaxin-1, a powerful eosinophil chemoattractant, are less susceptible to DSS-colitis and exhibit a reduced tumor burden following DSS+AOM treatment compared to wild-type mice [160].

In conclusion, neutrophils, MDSCs, and eosinophils play complex roles in both IBD and CAC. Understanding these role and the mechanisms by which these cells interact with other immune and non-immune cells in the gut is key to developing targeted therapies for preventing/treating CAC.

## 6. Natural Killer Cells, Natural Killer T Cells, and Innate Lymphoid Cells

NK cells are innate lymphocytes specialized in the detection and elimination of transformed cells through contact-dependent cytotoxicity and cytokine secretion. In response to IL-15 and IL-21, two cytokines over-produced in both IBD and CAC, these cells produce elevated levels of IFN-γ, which promotes the apoptosis of target tumor cells [41,161,162]. Notably, in cancer cells, the caspase-3-mediated cleavage of IL-18 generates a 15 kDa form of IL-18, referred to as short IL-18. Unlike mature IL-18, short IL-18 is not secreted, and moves into the nucleus, where it facilitates STAT1 phosphorylation at Ser727 and activates a signaling cascade, which enhances NK cell mobilization and cytotoxicity against tumors in CAC models [39].

NKT cells co-express both NK receptors and an invariant antigen receptor (T cell receptor; TCR) α-chain, which recognizes lipid antigens presented by the non-polymorphic CD1d molecule, unlike conventional T cells that recognize peptide-MHC complexes. CD1d-restricted NKT cells mainly consist of type I invariant NKT (iNKT) and type II NKT cells that react to glycolipids, α-galactosylceramide (α-GalCer) and sulfatide, respectively [163]. Upon activation, NKT cells quickly secrete large amounts of cytokines such as IFN-γ, IL-4, IL-10, IL-13, IL-17, and TNF-α, allowing them to shape innate and adaptive immune responses [164]. By engaging with CD1d-expressing non-immune cells (e.g., intestinal epithelial cells and enterochromaffin cells) and immune cells (e.g., monocytes, macrophages, innate lymphoid cells, and B cells), iNKT cells contribute to the maintenance of immune homeostasis in the intestine [165]. However, these cells can also exacerbate or regulate autoimmune and inflammatory diseases depending on the context. The human gut contains IL10-producing iNKT cells with suppressive capabilities towards pathogenic CD4+ T cells [166]. Consistently, iNKT cell-deficient mice display increased colitis severity, suggesting a predominantly protective function of colonic iNKT cells [165]. In contrast, type II NKT cells, which express high levels of CD161 and IL-13Rα, and produce high levels of IL-13 have been implicated in epithelial cytotoxicity and the atypical Th2 immune response characteristic of UC [167,168]. These findings indicate that type II NKT cells can be either protective or pathogenic in IBD but their role on the initiation and progression of CAC remains poorly understood. Studies in the AOM/DSS model indicate that the progression of colitis and CAC is highly dependent on the absence of iNKT cells [169], raising the possibility that these cell types exert mainly anti-tumoral properties in the colon.

Innate lymphoid cells (ILCs) are part of the same family as NK cells, but unlike NK cells, they do not have cytotoxic activity [170]. ILCs are primarily found in mucosal tissues, including the intestinal mucosa, where they play key roles in maintaining tissue integrity and immunity mainly through the production of cytokines and lipids [171]. ILCs are activated by stress-related signals in tissues, such as alarmins, cytokines, and other cell surface ligands/receptors that enable them to respond rapidly at the local level. There are three main subsets of ILCs: ILC1, ILC2, and ILC3, which are functionally similar to the Th cell subsets Th1, Th2, and Th17/Th22, respectively, and are involved in type 1, 2, and 3 immunity [172]. Recent studies in both mouse models and humans suggest that ILCs contribute to the development of sporadic CRC, with both pro-tumor and antitumor effects [173,174,175,176]. Research indicates that the frequency of intra-tumoral ILCs and the expression of specific ILC signature genes may serve as predictors for disease progression and response to PD-1 checkpoint inhibition therapy in CRC [177]. Although the contribution of ILCs in the development of CAC is not fully understood, evidence from animal models suggests that ILCs and especially ILC3s may contribute to CAC. During CAC, ILC3s are targeted by TNF-like cytokine 1A (TL1A), a member of the TNF superfamily, which specifically signals through its receptor, death receptor 3 (DR3) [44]. Genetic variants in TNFSF15, which encodes TL1A, confer increased risk for more severe forms of IBD [178,179]. Notably, overexpression of TL1A enhances the content of PCNA, β-catenin, c-myc, and Cyclin D1 in mice treated with AOM + DSS thereby increasing the development of CAC [180]. In this process, colonic tissue-resident ILC3s act as key sensors of TL1A signaling. Specifically, TL1A-stimulated ILC3s activate neutrophils and drive the expression of genes associated with neutrophils present in tumors. Moreover, studies in mice depleted of neutrophils support the role of these cells in the TL1A-mediated CAC [44]. ILC3s express high levels of nucleophosmin 1 (NPM1), a gene that is frequently mutated and associated with myelodysplastic syndrome and acute myeloid leukemia [181,182]. NPM1 interacts with various partners across different cellular compartments, including nucleolar factors, transcription factors, and histones [183]. Its presence in ILC3s is crucial for IL-22 production during DSS-induced colitis and TNBS-induced colitis. Additionally, mice with a deficiency of NPM1 in hematopoietic cells develop more tumors, with a larger size and greater tumor burden, compared to control mice [182]. The production of IL-22 by ILC3s, CD4+ T cells, and γδ T cells in the colon also depends on butyrophilin-like protein 2 (BTNL2). Mice lacking BTNL2 show reduced colonic tumorigenesis in response to AOM+DSS and exhibit more severe colitis symptoms compared to control mice, due to impaired IL-22 production. Similarly, blocking BTNL2 reduces colorectal tumor development in mice, and a recombinant mBTNL2-Fc protein proves to be therapeutic in DSS-induced colitis [184]. Another regulator of IL-22 by ILC3s is IL-17D, a member of the IL-17 family. IL-17D is expressed primarily by colonic epithelial cells, and IL-17D-deficient mice exhibit an impaired ILC3s-derived IL-22 production and are more susceptible to DSS-induced colitis and experimentally induced CAC than their wild-type counterparts [185]. IL-22-induced activation of STAT3 in intestinal epithelial cells leads to the upregulation of the oncostatin M (OSM) receptor, a cytokine member of the IL-6 family. In turn, OSM collaborates with IL-22 to maintain STAT3 activation in epithelial cells, promoting a pro-inflammatory epithelial response and enhancing immune cell recruitment to the inflamed intestine. Conditional deletion of the OSM receptor in intestinal epithelial cells protects mice from both colitis and CAC, while pharmacological inhibition of OSM reduces established CAC [45].

Altogether, the above data indicate that the innate immune cells orchestrate a dynamic response to tumorigenesis, with varying roles in tumor progression. These immune responses are further influenced by the complex interplay with adaptive immune cells.

## 7. Adaptive Immune Cells

Adaptive immune cells substantially contribute to the complexity of immune regulation in CAC. Among these, CD4^+^ Th lymphocytes, particularly Th1, Th2, and Th17 subsets, play pivotal but often opposing roles during inflammation-driven tumorigenesis, depending on their cytokine profile, activation state, and temporal involvement in disease progression [186]. In sporadic CRC, tumor-infiltrating CD4^+^ Th cells are generally associated with effective antitumor immunity and improved clinical outcomes [187]. In contrast, in IBD patients, CD4^+^ T cells are key drivers of chronic mucosal inflammation and might indirectly promote CAC development by sustaining a pro-tumorigenic inflammatory microenvironment [186]. However, studies in a model that mimics CD-associated CRC, based on intrarectal administration of TNBS combined with AOM, Osawa and colleagues demonstrated that IFN-γ–deficient mice developed significantly more colonic neoplasms than wild-type or IL-4-null mice. This increased tumor burden was accompanied by elevated expression of Th2-associated cytokines, including IL-4 and IL-5, suggesting that a Th2-skewed immune response may favor tumor growth. Beyond shaping the immune milieu, Th2 cytokines may directly contribute to tumor initiation, as these cytokines can induce expression of activation-induced cytidine deaminase in colonic epithelial cells, an enzyme capable of introducing DNA mutations. Moreover, the increased susceptibility of IFN-γ-deficient mice to CAC may also reflect impaired antitumor immune surveillance, as IFN-γ is a key activator of cytotoxic NK cells and CD8^+^ T lymphocytes [188,189]. Overall, these findings align with the results of our studies aimed at investigating the role of Smad7, an inhibitor of TGF-β1 signaling, in colon carcinogenesis [190]. Specifically, we showed that the number of Smad7-positive CD4+ T lymphocytes in the inflamed mucosa of IBD complicated by CAC was diminished as compared to that seen in the mucosa of uncomplicated IBD. In the murine model of AOM+DSS-induced CAC, mice over-expressing Smad7 in T cells and NKT cells developed a severe colitis characterized by a massive infiltration of the mucosa with CD8+ T cells and NKT cells and increased production of IFN-γ. However, those mice developed fewer tumors than control mice. The latter protective effects were dependent on IFN-γ, as deletion of the IFN-γ gene abolished the beneficial effect of Smad7-over-expressing T cells on CAC formation [191]. However, the relationship between Th1 immunity and CAC risk is not straightforward. Patients with colonic CD display elevated levels of Th1 cytokines yet remain at increased risk of developing CAC [192]. Importantly, when disease extent and duration are comparable, the risk of CAC in CD is similar to that observed in UC, which is not classically associated with a Th1-type immune response [193]. This apparent paradox has been clarified by advances in the understanding of CD pathogenesis, particularly the recognition of the IL-23/Th17 axis as a central inflammatory pathway [194]. CD was historically considered a Th1-mediated disorder based on early studies demonstrating the pathogenic role of IL-12 and IFN-γ [195]. Neutralization of the p40 subunit, which is shared by IL-12 and IL-23, ameliorated intestinal inflammation in both experimental colitis and CD patients [196,197,198]. The subsequent discovery that IL-23 consists of p40 paired with a distinct p19 subunit shifted attention toward IL-23 as a key pathogenic cytokine in both CD and UC. This was supported by findings showing reduced colitis severity following IL-23p19 blockade in IBD patients and in mice and by the strong genetic association between IL-23 receptor (IL-23R) polymorphisms and CD [199,200,201]. IL-23 is now known to be essential for the maintenance and pathogenicity of Th17 cells [202]. Th17-related cytokines, including IL-17A, IL-21, and IL-22, are abundantly produced in the inflamed gut of CD patients and exert pleiotropic effects. While these cytokines can contribute to epithelial repair and barrier integrity, they also display potent pro-inflammatory and mitogenic properties that may support tumor development [203,204,205]. Importantly, Th17 cells represent a heterogeneous and highly plastic population. Subsets of Th17 cells isolated from the intestinal mucosa of CD patients co-produce IL-17A and IFN-γ, and are highly responsive to IL-23 [206]. IL-21 further modulates this balance by influencing the differentiation and stability of both Th1 and Th17 lineages [41]. Elevated levels of IL-21 have been detected in the intestinal mucosa of patients with UC-associated CAC and in mice with AOM+DSS-induced CAC. In this experimental model, IL-21-deficient mice developed a markedly attenuated form of colitis compared with wild-type animals, as evidenced by reduced epithelial damage, decreased T-cell infiltration, and lower production of pro-inflammatory cytokines such as IL-6 and IL-17A. Consistent with the reduced inflammatory response, IL-21 knockout mice also exhibited a lower tumor burden, with fewer and smaller colonic tumors than control mice. Mechanistic analyses indicated that IL-21 promotes the recruitment of CD4^+^ T cells to tumor and peritumoral regions and amplifies IL-6 and IL-17A production, leading to enhanced STAT3 signaling. IL-6 is made by additional immune and non-immune cells and targets cancer cells thus sustaining the activation of STAT3 and, eventually, cell growth [207]. Administration of a neutralizing IL-21 antibody to WT mice after the last DSS cycle decreased the tumor burden, thus suggesting that the tumor promoting effect of IL-21 in this model is not entirely dependent on the inhibition of inflammation [208]. In addition to directly regulating immune responses, IL-21 may thus indirectly influence tumor development by targeting non-immune cells. This aligns with the demonstration that IL-21 induces stromal cells to produce matrix metalloproteinases [209], and other molecules that control the various phases of CAC [210]. Fichtner-Feigl and colleagues demonstrated that IL-21 drives colitis-associated tumorigenesis by stimulating tumor cell proliferation and impairing the antitumor activity of CD103^+^CD8^+^ cytotoxic T lymphocytes. In agreement with these findings, Jauch et al. reported that IL-21 deficiency results in reduced IL-17A expression, increased IFN-γ levels, diminished epithelial cell proliferation, and enhanced epithelial apoptosis in intestinal tumors following AOM+DSS treatment [211]. The increased risk of CAC observed in subsets of CD patients and UC patients may therefore reflect context-dependent alterations in Th17 cell-derived cytokines at the mucosal level [190,212].

CD8^+^ cytotoxic T lymphocytes represent immune subsets with intrinsic antitumor potential [213]. Their contribution to cancer immunity extends beyond direct killing of transformed cells and encompasses broader regulatory functions that can either restrain or promote tumor development depending on the inflammatory context. CD8^+^ T cells play a central role in immune surveillance by recognizing tumor-associated antigens presented by antigen-presenting cells and executing cytotoxic programs mediated by perforin, granzymes, Fas ligand, and TRAIL. Activated CD8^+^ T cells also produce IFN-γ, which amplifies antitumor immunity by enhancing antigen presentation, activating innate immune cells, and directly inhibiting tumor cell proliferation [214]. In sporadic CRC, high infiltration of CD8^+^ T cells is consistently associated with favorable prognosis [215]. In CAC, however, the role of CD8^+^ T cells is more complex and remains controversial. Comparative analyses of CAC versus sporadic CRC have revealed increased CD8^+^ T cell infiltration in inflammation-associated tumors without a corresponding survival benefit [112,216,217]. This discrepancy likely reflects the dual role of CD8^+^ T cells in IBD, where they contribute not only to tumor immune surveillance but also to epithelial injury and chronic inflammation. In active UC and CD, mucosal CD8^+^ T cells express high levels of perforin and granzymes, promoting epithelial cell death and barrier disruption [218]. While epithelial destruction does not directly cause cancer, it triggers compensatory epithelial proliferation in a genotoxic inflammatory environment, thereby increasing the likelihood of malignant transformation [52,219]. Consistent with this interpretation, perforin-deficient mice subjected to the AOM/DSS model of CAC develop less severe colitis and significantly fewer tumors than wild-type animals [220]. Conversely, experimental conditions that intensify cytotoxic immune responses within tumors can be protective. Regulatory T cells (Tregs), defined by expression of CD4, CD25, and the transcription factor Foxp3, are critical modulators of immune tolerance and inflammation [221]. Tregs exert immunosuppressive effects through direct cell–cell interactions and the secretion of anti-inflammatory cytokines such as IL-10 and TGF-β [222]. While these functions are essential for preventing autoimmunity and limiting tissue damage, they may also compromise antitumor immune surveillance.

In established cancers, Tregs are generally considered pro-tumorigenic, and high Foxp3 expression has been associated with poor prognosis in several malignancies [223]. In CRC, however, Treg infiltration paradoxically correlates with improved clinical outcomes [224]. The role of Tregs in CAC remains incompletely understood. Given their potent anti-inflammatory properties, Tregs may protect against inflammation-driven tumor initiation by limiting epithelial damage and genotoxic inflammatory signaling. In line with this hypothesis, mice deficient in Runx3, a transcription factor essential for Treg differentiation and function, develop more severe colitis and are more susceptible to inflammation-associated colon tumors [225]. These findings suggest that Tregs can exert antitumor effects in CAC by dampening chronic inflammation. Nevertheless, once neoplastic lesions are established, Treg-mediated immunosuppression may hinder effective antitumor immune responses, potentially facilitating tumor progression. Thus, the impact of Tregs in CAC is likely stage-dependent, underscoring the need for temporal and context-specific therapeutic strategies targeting this population [226]. B cells also emerge as interesting players in CAC. Comparative studies of CAC versus sporadic CRC have shown that dense infiltrates of CD20^+^ B cells correlate with improved survival in CAC, suggesting a potentially protective effect [216]. These B cells may mediate antitumor activity via antibody production, antigen presentation, or cytokine secretion [227]. For instance, B cell-derived IL-10 is essential to limit pathogenic Th1/Th17 T cell responses during chronic colitis, while IgA PCs derived from IL-10+ B cells are being implicated in restraining tumorigenesis during CAC. Formation of a tumor-protective intestinal environment has been associated with clonal expansion of specific types of colonic IgA PCs and development of an altered microbiota that attenuated CAC [227]. Notably, in IBD mucosa, CD19^+^ plasma cells can produce granzyme B, which has been shown to kill CRC cells in vitro [228]. This aligns with findings indicating that CD19^+^ B cells can suppress tumor aggressiveness in various cancers, such as triple-negative and HER2-positive breast cancers [229]. Furthermore, CD19^+^ B cells have been shown to enhance antitumor immunity in muscle-invasive bladder cancer [230]. In this context it is noteworthy that CD19 chimeric antigen receptor (CAR) T-cell therapy has been recently explored as a treatment for refractory UC, even though it remains to be ascertained whether depletion of B cells can eventually enhance the risk of CAC [231,232].

In conclusion, adaptive immune cells play critical and complex roles in the immune regulation of CAC, depending on their activation state and timing.

## 8. Potential Drugs for the Treatment of Colitis-Associated Colorectal Cancer

In recent years, extensive research has focused on evaluating the potential effectiveness of new compounds in the prevention/treatment of CAC using preclinical models. One such compound is Celastrol, a pharmacologically active triterpene derived from the traditional Chinese medicinal plant Tripterygium wilfordii Hook F. Early studies have demonstrated that Celastrol alleviates DSS-induced colitis in mice by regulating intestinal epithelial homeostasis, reducing oxidative stress in the colon, and decreasing levels of inflammatory cytokines [233]. Furthermore, Celastrol induced apoptosis in human CRC cells by enhancing the expression of death receptors and activating the β-catenin signaling pathway, while also inhibiting the invasiveness of CRC cells [234]. Lastly, studies have shown that Celastrol notably decreased the number of colonic neoplasms and the tumor area, while also enhancing the survival rate of mice with AOM/DSS-induced CAC [235]. Another promising anti-cancer compound is silibinin, a natural polyphenolic flavonoid derived from the milk thistle plant [236]. In a chemopreventive model, oral administration of silibinin to AOM/DSS-treated mice reduced colitis induction and the progression of CAC. Additionally, silibinin treatment lowered IL-6 production and inhibited STAT3 activation in intestinal tumor cells, thereby suppressing tumor cell proliferation and promoting apoptosis [237]. Additional mechanistic studies showed that silibinin caused cell cycle arrest at the G2/M phase in cancer cells by decreasing the expression of Cdc25C and inhibiting the dephosphorylation of CDK1 at multiple sites [238].

Andrographolide, a natural diterpenoid and the primary active compound found in Andrographis paniculata, a plant native to Southeast Asia. Andrographolide significantly protected mice from CAC in the AOM-DSS mouse model by inhibiting NLRP3 inflammasome activation in macrophages. Additional studies revealed that Andrographolide induced mitophagy by suppressing the PIK3CA-AKT1-MTOR-RPS6KB1 pathway, which restored mitochondrial membrane potential and inactivated the NLRP3 inflammasome [239].

Thalidomide, a synthetic derivative of glutamate, is a strong inhibitor of NF-kB activation and has been used to treat active phases of IBD [240]. Furthermore, in the AOM/DSS-induced CAC model, thalidomide treatment decreased both the incidence and size of tumors [241].

Recent studies have demonstrated that orally administered nanotherapeutics containing water-insoluble curcumin and 7-ethyl-10-hydroxycamptothecin, compounds with anti-inflammatory and cytotoxic properties, respectively, accumulated in the inflamed intestinal regions and tumor tissues of mice with AOM/DSS-induced CAC. This accumulation resulted in a significant reduction in tumor burden [242]. These findings align with previous studies reporting the anti-cancer effects of curcumin in the AOM+DSS-induced CAC [243,244]. In line with data supporting a pro-tumorigenic effect of STAT3 signaling in the colon, TTI-101, a small-molecule STAT3 inhibitor, was found to be beneficial in the AOM+DSS-induced CAC model [245].

Immune checkpoint inhibitors (ICIs) represent an effective therapeutic approach for a range of cancers, including specific subtypes of sporadic CRC, particularly those with microsatellite instability. These treatments work by targeting immune checkpoint proteins, such as programmed cell death protein 1 (PD-1) and its ligand PD-L1, or cytotoxic T-lymphocyte-associated protein 4 (CTLA-4), thereby stimulating the body’s immune response [246]. Anti-PD-L1 nanobodies are effective in reducing the tumor burden in mice with AOM+DSS-induced CAC [247]. However, the potential effectiveness of these drugs must be weighed against their possible adverse effects, particularly immune-mediated colitis [248].

While the exact pathogenesis of CAC is not yet fully understood, the burden of colonic inflammation plays a crucial role in the development of this neoplasia. Consequently, drugs designed to induce and maintain remission in IBDs could simultaneously serve as effective chemopreventive agents for CAC, as supported by studies in preclinical models [249,250]. However, future clinical trials are needed to address this issue in human IBD.

## 9. Conclusions

Epidemiological evidence clearly indicates that, in IBD patients, the duration and the extent of intestinal inflammation are major determinants of colorectal tumorigenesis [1,3,4,5]. At the same time, it has become increasingly evident that tumor-promoting inflammation and protective antitumor immunity coexist within the intestinal microenvironment of CAC. The immune system therefore represents a double-edged sword: immune-mediated tissue injury and pro-inflammatory cytokine signaling can drive epithelial transformation and tumor growth, while immune surveillance mechanisms mediated by cytotoxic lymphocytes, NK cells, and specific macrophage subsets can restrain malignant progression. The overall outcome appears to depend on a complex interplay between immune-cell composition, cytokine networks, microbial signals, and metabolic cues within the intestinal mucosa.

An important emerging concept is that immune-cell plasticity and temporal context critically determine the functional consequences of inflammation. Many immune populations described in this review, including macrophages, neutrophils, and T cell subsets, can exert both tumor-promoting and tumor-suppressive activities depending on their activation state and the stage of disease. Consequently, CAC development cannot be explained by the presence of specific immune populations alone but rather by the dynamic balance between pro-tumorigenic and antitumor pathways operating over time. Future research should therefore move beyond static descriptions of immune infiltration and instead focus on the spatiotemporal dynamics of immune responses during the transition from chronic inflammation to dysplasia and carcinoma. Advances in single-cell transcriptomics, spatial transcriptomics, and high-dimensional immunophenotyping are likely to provide critical insights into how immune cell states evolve during this process and how they interact with epithelial, stromal, and microbial components of the intestinal microenvironment.

Another important direction for future investigation involves the integration of immune, microbial, and metabolic pathways in CAC pathogenesis. Increasing evidence indicates that microbial products and dysbiosis influence innate immune receptors such as TLRs, thereby shaping inflammatory circuits that modulate tumor development [251]. In parallel, metabolic programs within immune cells (e.g., pathways controlling polyamine synthesis, NAD^+^ metabolism, and hypoxia signaling) have emerged as key regulators of macrophage polarization and immune suppression within tumors [252,253,254,255,256]. Understanding how these metabolic checkpoints intersect with microbial and cytokine signaling may reveal new opportunities for therapeutic intervention aimed at restoring protective immune responses while limiting chronic inflammatory damage.

From a translational perspective, a deeper understanding of immune regulation in CAC may enable the development of biomarkers capable of identifying IBD patients at highest risk for malignant transformation. Recent studies have demonstrated that serum concentrations of short-chain N-acyl homoserine lactones (scAHLs), a group of bacterial quorum-sensing molecules, are elevated in patients with UC compared to healthy individuals. Notably, the highest scAHL levels were observed in UC patients with active inflammation and a disease duration of 10 years or more, suggesting a potential link to colon tumor development. In line with this, systemic administration of C6-scAHL to mice was found to exacerbate tumorigenesis induced by AOM+DSS [257]. Along the same line is the demonstration that trimethylamine n-oxide, a metabolic product derived from the gut microbiota, promotes inflammation-mediated colorectal carcinogenesis induced by AOM+DSS by enhancing Wnt signaling [258].

While these findings outline a potential link between microbiota and CAC, additional research is required to determine whether the levels of these metabolites could serve as a marker to identify patients at higher risk for developing CAC.

Current surveillance strategies rely primarily on disease duration and anatomical extent of inflammation [6,7,8,9,10,11]; however, these parameters fail to fully explain the heterogeneity in cancer risk among patients with long-standing disease. Immune-based biomarkers, such as patterns of neutrophil infiltration, macrophage polarization states, cytokine signatures, or the presence of immunosuppressive cell populations, may help refine risk stratification and guide personalized surveillance programs. In addition, integrating immune profiling with emerging molecular markers of epithelial stress or genomic instability could significantly improve early detection of dysplasia.

Therapeutically, targeting immune pathways involved in CAC represents an attractive but challenging strategy. The dual role of many immune mediators suggests that selective immune modulation rather than broad immunosuppression will be necessary to achieve effective cancer prevention without compromising mucosal defense. Approaches aimed at restoring immune equilibrium (e.g., reprogramming TAMs toward antitumor phenotypes), enhancing cytotoxic lymphocyte activity, or promoting the resolution of neutrophil-driven inflammation may represent promising strategies. Moreover, interventions targeting upstream regulators of inflammation, including microbial sensing pathways or metabolic checkpoints within immune cells, could potentially prevent the establishment of a tumor-permissive microenvironment in the chronically inflamed colon.

Finally, translating these findings into clinical practice will require carefully designed longitudinal studies and translational clinical trials in well-characterized IBD cohorts. Combining advanced immunological profiling with clinical outcomes, endoscopic surveillance data, and microbiome analysis may allow the identification of actionable pathways that drive CAC in humans. Ultimately, integrating immunology, microbiology, and epithelial biology will be essential for developing precision strategies aimed at preventing or intercepting cancer development in patients with IBD.

## Figures and Tables

**Table 1:** Cells source and regulatory effects of cytokines on tumorigenesis.

Cell Source	Tumor-Promoting Cytokines	Tumor-Suppressing Cytokines
M1 macrophages	TNF-α, IL-1β, IL-6, IL-23	IL-12, Type I IFNs, IL-1β, IL-23
M2 macrophages	IL-10, TGF-β, IL-6, TGF-β, IL-34, IL-1β, TNF-α, TNSF14	-
Neutrophils	IL-1β, IL-8, TNF, IL-17	-
NK cells	-	IFN-γ
NKT cells	IL-4, IL-13, IL-10, IL-17, TNF-α	IFN-γ, IL-10
MDSCs	IL-17	-
ILCs	IL-22, TL1A	IL-22
Epithelial & stromal cells	IL-6, IL-8, OSM, IL-34	IL-17D
Th1 CD4^+^ T cells	-	IFN-γ
Th2 CD4^+^ T cells	IL-4, IL-5, IL-13	-
Th17 CD4^+^ T cells	IL-17A, IL-21, IL-22	-
B cells	IL-10	IL-10
CD8^+^ T cells	-	IFN-γ
Regulatory T cells	IL-10, TGF-β	IL-10, TGF-β

Abbreviations: IL, interleukin; TNF, tumor necrosis factor; TNSF, tumor necrosis factor superfamily member; OSM, oncostatin; TL1A, TNF-like ligand; IFN, interferon; TGF, transforming growth factor.

## Data Availability

The original contributions presented in this study are included in the article. Further inquiries can be directed to the corresponding author.
